# Genomic characterization of multidrug-resistant *Escherichia albertii* of fish origin—first isolation and insights into a potential food safety threat

**DOI:** 10.3389/fmicb.2025.1521202

**Published:** 2025-02-27

**Authors:** Kandhan Srinivas, Sandeep Ghatak, Arockiasamy Arun Prince Milton, Samir Das, Kekungu-u Puro, Daniel Aibor Pyngrope, Madesh Angappan, Mosuri Chendu Bharat Prasad, Dadimi Bhargavi, Nur Abdul Kader, Vanita Lyngdoh, Heiborkie Shilla, John Pynhun Lamare

**Affiliations:** ^1^Division of Veterinary Public Health, ICAR – Indian Veterinary Research Institute, Bareilly, India; ^2^Division of Animal and Fisheries Sciences, ICAR Research Complex for NEH Region, Umiam, India

**Keywords:** *Escherichia albertii*, fish, first report, MDR, virulence, genomics

## Abstract

**Introduction:**

*Escherichia albertii* is an emerging food-borne pathogen with zoonotic potential which is often under-reported due to misidentifications.

**Materials and methods:**

The current study identified *E. albertii* from retail fish sold in market which was confirmed by phenotypic (colorless colonies on Xylose-Rhamnose-Melibiose MacConkey Agar), genotypic (dual target uniplex PCR-based detection) and genomic methods (CheckM analysis). In this paper we report the phenotypic characters of the isolate and genomic features such as resistome, virulome and mobilome followed by *in silico* O and H antigen based typing and comparative phylogenomics using various tools (RAST, RGI v6.0.0, ABRicate v1.0.1, PathogenFinder v1.1, PlasmidFinder v2.0, BacAnt v3.3.1, Phigaro v2.4.0, MAFFT v7.490, FigTree v1.4.4).

**Results and discussion:**

Multidrug resistance was identified with reduced susceptibility to gentamicin, azithromycin, ceftazidime and cefotaxime with a Multiple Antibiotic Resistance (MAR) index of 0.33. Clinically important virulence genes such as *eae, cdt, east1* formed a part of the virulome and the probability of being pathogenic to humans was found to be 0.883. The genome was found to harbor mobile genetic elements such as plasmids [IncFIA, IncFIB(pB171), IncFII(pSE11)], transposons (Tn*3411*, Tn*6292*) and prophages (Siphoviridae, Myoviridae, Podoviridae). Various typing methods such as biotyping, multilocus sequence typing and *in silico* O and H antigen typing classified the isolate into biotype 3, multi locus sequence type 4596, O-genotype 4 and H-genotype 1. Phylogenomically, the isolate was placed close to isolate from neighboring country of China. Identification of virulent multidrug-resistant *E. albertii* from new food source such as fishes increases the risk for fish eating population and necessitates the requirement of further elucidation and development of appropriate control strategies.

## 1 Introduction

*Escherichia albertii* has emerged as a potentially important food-borne pathogen over the last few years (Masuda et al., 2020; Hirose et al., 2022; Muchaamba et al., 2022). Since its initial identification from diarrhoeagenic infant patients from Bangladesh, the hazard level has steadily ascended over the years (Huys et al., 2003; Muchaamba et al., 2022). Recent reports have hinted that the virulence potential of *E. albertii* could be on par with *Escherichia coli* due to shared repertoire of genes and subsequentially it has been implicated in clinical manifestations in humans as well (Bours et al., 2010; Luo et al., 2021). Frequent association of the pathogen with various clinical conditions affecting humans such as diarrhea and urinary tract infections over a wide geographical area has gradually raised global concerns associated with this pathogen (Bours et al., 2010; Brandal et al., 2015; Zaki et al., 2022; Iguchi et al., 2023). The recent reports have showcased the ability of *E. albertii* to harbor diverse clinically important virulence genes such as intimin, cytolethal distending toxin, Shiga toxin and Enteroaggregative *E. coli* heat-stable enterotoxin 1 (EAST1) which often cause gastro-intestinal disorders in humans (Luo et al., 2021; Muchaamba et al., 2022).

The resistances profile of *E. albertii* have leaped from reporting susceptible patterns to a multidrug-resistant strains with inclusion of resistance against clinically important drugs such as carbapenem (Wang et al., 2022) and colistin (Sonnevend et al., 2022). Genotypically, they were also found to carry Extended Spectrum Beta Lactamase (ESBL) genes such as *bla*_TEM-141_ and *bla*_CTX-M-55_ (Guo et al., 2024) as well as mcr genes responsible for colistin resistance (Li et al., 2018; Sonnevend et al., 2022). Mobile genetic elements such as transposons, plasmids, phages and integrons play an important role in the horizontal transfer and further dissemination of antimicrobial resistance genes and *E. albertii* is no exception (Partridge et al., 2018). Various typing methods have been used to study the epidemiology of *E. albertii* which include the conventional biotyping (Murakami et al., 2019), Multilocus sequence typing (MLST) (Luo et al., 2021) and pulsotyping (Leszczyńska et al., 2023). In addition, PCR based methods harnessing the variations in repetitive elements interspersed between the genes have also been used (Grillová et al., 2018).

Food and water as a vehicle for the transmission of *E. albertii* was proven in a series of outbreaks in Japan (Masuda et al., 2020). Additionally, *E. albertii* has also been isolated from foods of plant as well as animal origin. Plant based sources include lettuce, Japanese parsley, Mitsuba, Mizu, watercress and cucumber; whereas, chicken, pork, milk, cheese, mutton, duck meat, Japanese rock oyster and Pacific oyster (Fiedler et al., 2018; Arai et al., 2022; Hirose et al., 2022; Muchaamba et al., 2022). Additionally, the organism has also been spotted in river water and drinking water systems (Fiedoruk et al., 2014; Maheux et al., 2014).

With this background, the current study aims to document the first report of the isolation of *E. albertii* from fish sold in the retail markets. Further we attempted to characterize the genome of the isolate in terms of resistance genes, virulence genes, mobile genetic elements, *in silico* typing and phylogenomic positioning with respect to global dataset of genomes to obtain cues on the transmission and genetic diversity.

## 2 Materials and methods

### 2.1 Isolate identification

Single ichthyic isolate of *E. albertii* (Ea_FM1) was recovered from fresh fish meat sample collected from Laitumkhrah market of Meghalaya as part of routine surveillance work of food samples in the laboratory (Institutional Animal Ethics Committee clearance no. V-11011(13)/12/2023-CPCSEA-DADF dated December 5, 2023). The sample (25 g) was enriched in novobiocin (HiMedia, Mumbai, India) supplemented modified EC broth (HiMedia, Mumbai, India) incubated at 42°C for 24 h (Arai et al., 2021) followed by plating onto modified Xylose-Rhamnose-Melibiose MacConkey (XRM-MacConkey) agar (MacConkey agar base; D-xylose [HiMedia, Mumbai, India]; L-rhamnose [Sisco Research Laboratories, Mumbai, India]; D-(+)-Melibiose [Sigma-Aldrich, MA, USA]) incubated at 37°C for 48 h (Hinenoya et al., 2020). Colorless colonies were later confirmed with the help of combination of conventional PCRs targeting a 393 bp region of DNA binding transcriptional activator of cysteine biosynthesis gene (Lindsey et al., 2017) and a 449 bp region of *E. albertii* specific cytolethal distending toxin (*Eacdt*) gene (Hinenoya et al., 2019).

### 2.2 Phenotypic characterization

Phenotypic characterization involved the assessment of antimicrobial susceptibility profile using CLSI guidelines (Clinical and Laboratory Standards Institute, 2023). The isolate was pitted against 12 clinically important antibiotics namely ampicillin (10 mcg), cefotaxime (30 mcg), ceftriaxone (30 mcg), cefoxitin (30 mcg), ceftazidime (30 mcg), imipenem (10 mcg), gentamicin (10 mcg), azithromycin (15 mcg), tetracycline (30 mcg), ciprofloxacin (5 mcg), co-trimoxazole (25 mcg) and chloramphenicol (30 mcg) belonging to various antimicrobial classes. Interpretative criteria for *Enterobacteriaceae* as stipulated by Clinical and Laboratory Standards Institute were followed (Clinical and Laboratory Standards Institute, 2023). Resistance to 3 or more different classes of antibiotics was taken as the criteria to confer multidrug resistance (Magiorakos et al., 2012). MAR index of the isolate was calculated as described earlier (Krumperman, 1983). Biofilm forming ability of the isolate was also tested by microtiter plate assay using previously established methods with *Acinetobacter baumannii* (ATCC 19606) and *E. coli* DH5α (New England Biolabs) as positive and negative control, respectively (Srinivas et al., 2023). Motility assay was performed as by stabbing onto semisolid Tryptone Soy agar containing 0.35% agar and incubation for 37°C. Hemolysis property was examined by streaking onto 5% sheep blood agar with *Staphylococcus aureus* (ATCC 25923) and *E. coli* (ATCC 25922) as positive and negative control, respectively. Biotyping of *E. albertii* isolate was undertaken with the help of an array of biochemical tests (Indole production and lysine decarboxylase) as specified earlier (Murakami et al., 2019).

### 2.3 Sequencing and assembly

Whole genome sequencing of the isolate was outsourced to M/S Eurofins Genomics India Pvt. Ltd. (Bengaluru, India). Illumina HiSeq platform was used for paired-end sequencing. The reads were scrutinized for quality using FastQC tool with default settings (Andrews, 2010). *De novo* assembly of the genome was undertaken with the help of Shovill tool ver. 1.0.9 with Spades assembler after triggering the following switches/parameters: “-trim,” “read error” and “post-assembly correction” (Seemann, 2018). Taxonomy identification was done using CheckM (Wood et al., 2019).

### 2.4 Public dataset and reference genome

*E. albertii* genomes submitted to NCBI were downloaded for phylogenetic analysis. From a collection of 672 genomes downloaded from NCBI (09 April 2024) a total of 50 representative genomes were selected based on mash distances calculated through Assembly Dereplicator tool v0.3.2.^1^ A distance of 0.001 with a cut off value of 50 genomes was used for downsizing number of genomes. The genomes were coded in X_Y_Z format for ease of downstream processing, where X stands for country, Y stands for isolation source and Z stands for isolate serial number (Supplementary Table 1).

### 2.5 Genome annotation and mapping

The Ea_FM1 genome was annotated employing online service from RAST (Rapid Annotation using Subsystems Technology) with default settings (Keegan et al., 2016). Genome map was also constructed using BLAST Ring Image Generator (BRIG) with preset parameters (Alikhan et al., 2011). Nucleotide polymorphisms were identified using Snippy tool v.4.6.0 (Seemann, 2018). *E. albertii* KF1 genome (CP007025) was used as reference for Snippy and BRIG analysis.

### 2.6 Resisto-virulo-mobilome analysis

Resistome was determined using RGI tool v6.0.0 employing Comprehensive Antimicrobial Resistance Database (CARD) v3.2.5 (Alcock et al., 2020). The switches “perfect” and “strict” cut-offs were triggered to obtain high stringency results with >95% similarity. Virulome was determined using ABRicate tool v.1.0.1^2^ with VFDB database updated till 14 August 2024. Sequence similarity and minimum coverage criteria were set at 75 and 80%, respectively. Pathogenic potential of the isolate toward humans were computed mathematically with the help of PathogenFinder v1.1 (Cosentino et al., 2013), an online service hosted by Center for Genomic Epidemiology.^3^ Mobilome (plasmids, transposons and integrons) were ascertained with the help of multiple tools. Plasmids harbored in the genome were detected using online PlasmidFinder 2.0 server provided by Center for Genomic Epidemiology.^4^ BacAnt v3.3.1 was used to determine the presence of transposons and integrons searched against TransposonDB v.2.0 and IntegronDB v.2.0, respectively (Hua et al., 2021). Minimum criteria for sequence identity and coverage threshold were set at 90 and 80%, respectively. Prophages inserted into the genome were identified with the help of Phigaro v.2.4.0.^5^ The details related to virus orthologous groups (VOGs) were obtained from VOGDB.^6^

### 2.7 *In silico* typing

*In silico* typing of the isolate was undertaken employing Multi locus sequence typing (MLST) scheme of *E. coli* (Wirth et al., 2006). Online MLST 2.0 tool provided by CGE was used for MLS typing of the isolate.^7^ Further, *E. albertii*-specific *in silico* typing based on antigenic diversity of somatic and flagellar antigens as described earlier were also followed (Ooka et al., 2019; Nakae et al., 2021).

### 2.8 Phylogenomic analysis

To get insights on the phylogenomic positioning of the isolate in relation to other global isolates, the ichythic isolate along with the reference genome and 50 other representative genomes chosen as mentioned earlier were included in a phylogenomic analysis. The core genome alignment was accomplished with MAFFT v7.490 with ‘auto’ option (Katoh and Toh, 2008). ModelFinder algorithm in IQ-TREE v2.0 was used to identify the suitable model with the least Bayesian Information Criterion (BIC) score (Kalyaanamoorthy et al., 2017). For identification of the clusters, the TreeLink tool (Allende et al., 2015) was used. The final tree was visualized with the help of FigTree v1.4.4.^8^

## 3 Results

### 3.1 Isolation and identification of *E. albertii*

**Figure 1:**
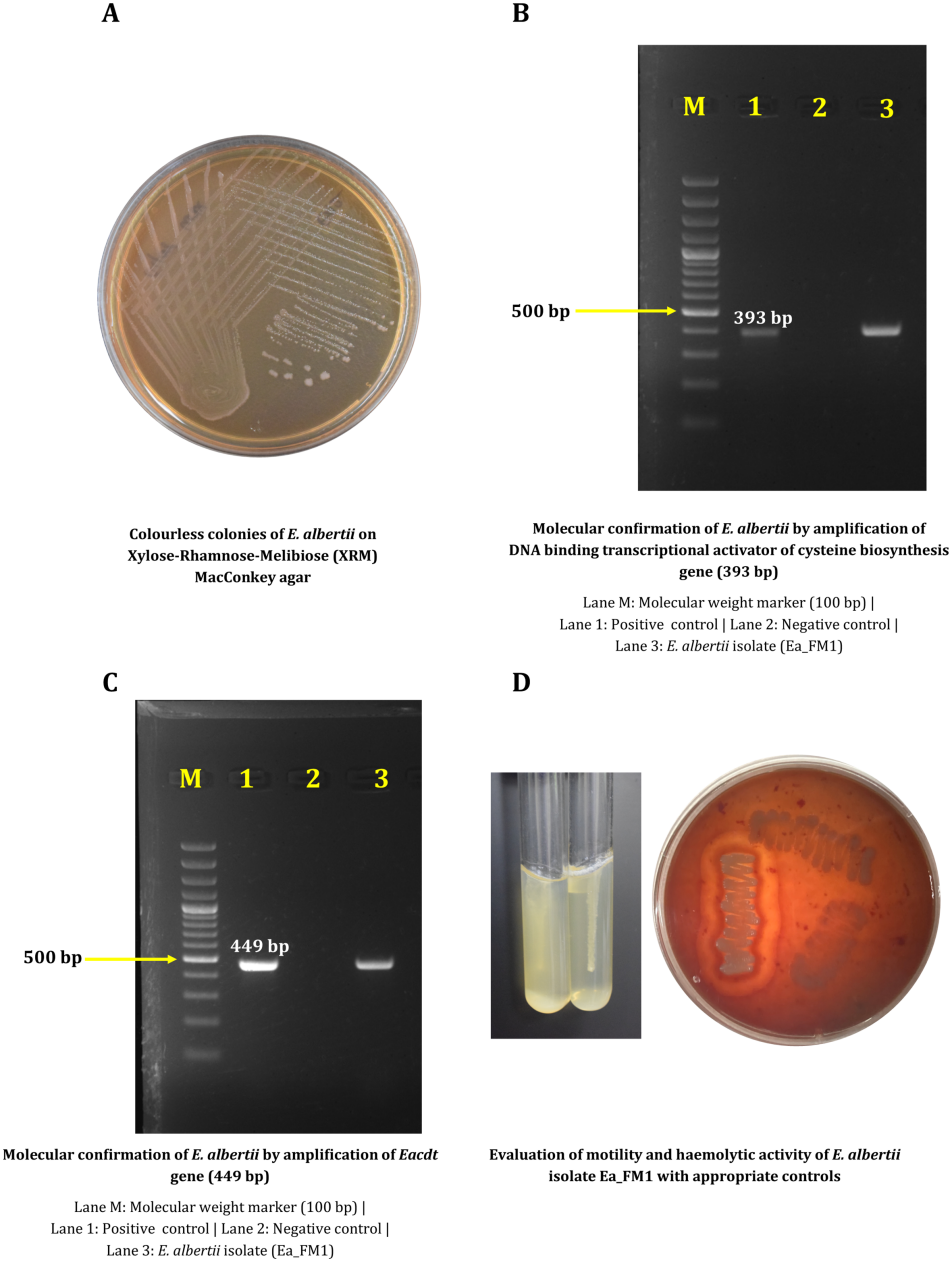
Phenotypic and molecular characterization of *Escherichia albertii* Ea_FM1 isolate. **(A)** Colorless colonies of *E. albertii* on Xylose-Rhamnose-Melibiose (XRM) MacConkey agar. **(B)** Molecular confirmation of *E. albertii* by amplification of DNA binding transcriptional activator of cysteine biosynthesis gene (393 bp). **(C)** Molecular confirmation of *E. albertii* by amplification of *Eacdt* gene (449 bp). **(D)** Evaluation of motility and haemolytic activity of *E. albertii* isolate Ea_FM1 with appropriate controls.

The ichthyic isolate Ea_FM1 was recovered from fish meat sample and was confirmed by phenotypic and molecular methods. Colorless colonies were observed on XRM MacConkey agar (Figure 1A) which was further confirmed by two different molecular diagnostic targets yielding the respective amplicons on gel electrophoresis (Figures 1B,C).

### 3.2 Antimicrobial susceptibility typing and biofilm forming ability

Antimicrobial susceptibility testing against 12 different antibiotics revealed resistance against gentamicin, cefotaxime, ceftriaxone and azithromycin by virtue of which the isolate is multidrug-resistant with a MAR index of 0.33 (Table 1). Experiment to assess the biofilm forming ability revealed the negligible ability of the isolate to form biofilm in comparison to *E. coli* DH5α. Other phenotypic characteristics include the inability to be motile at 37°C and the inability to cause hemolysis on blood agar (Figure 1D). Biotyping based on biochemical reactions categorized the isolate into Biotype 3 (Lysine and Indole positive).

**Table 1:** Phenotypic characteristics of *Escherichia albertii* isolate Ea_FM1.

Isolate	Antimicrobial susceptibility testing												Biofilm forming ability	Haemolysis	Motility	Biotyping	MAR index
	AMP (10)	GEN (10)	CIP (5)	TE (30)	COT (25)	CE (30)	CX (30)	CAZ (30)	IPM (10)	CTR (30)	C (30)	AZM (15)					
Ea_FM1	20 (S)	14 (R)	24 (I)	24 (S)	23 (S)	22 (R)	17 (I)	19 (I)	20 (I)	19 (R)	18 (S)	10 (R)	Negative	Negative	Negative	Biotype 3	0.33

### 3.3 Genome features

**Figure 2:**
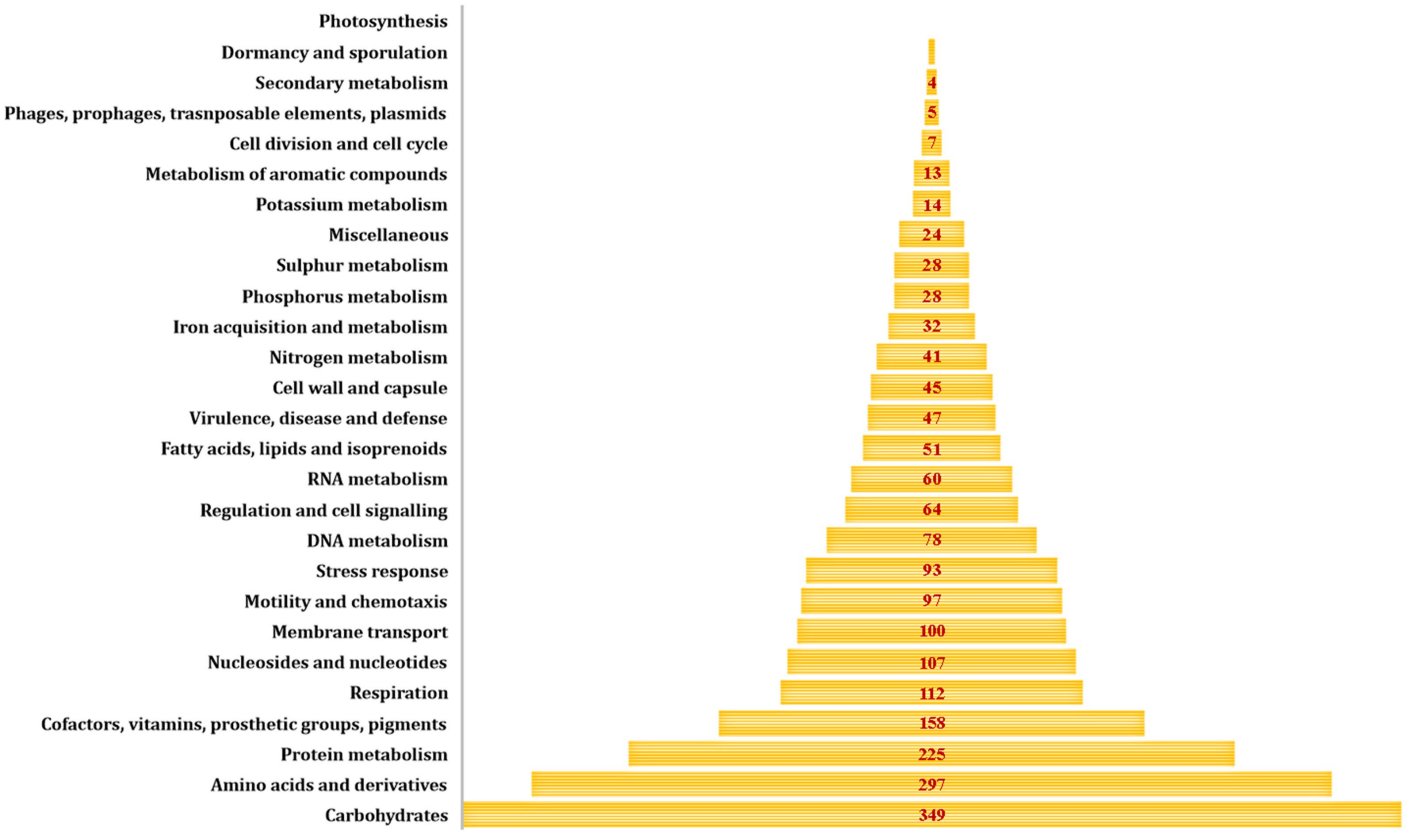
Classification of coding sequences according to RAST annotation of Ea_FM1 genome.

The size of the draft genome was 4.7 Mb with a G + C content of 49.5% (Figure 2). Number of contigs were 111 with a coverage of 11×. Taxonomy check using CheckM confirmed the identification of the isolate as *E. albertii*. Genome sequence was submitted to NCBI under the accession number JBBEFQ000000000.1 under BioProject: PRJNA889995.

### 3.4 RAST annotation and snippy analysis

**Figure 3:**
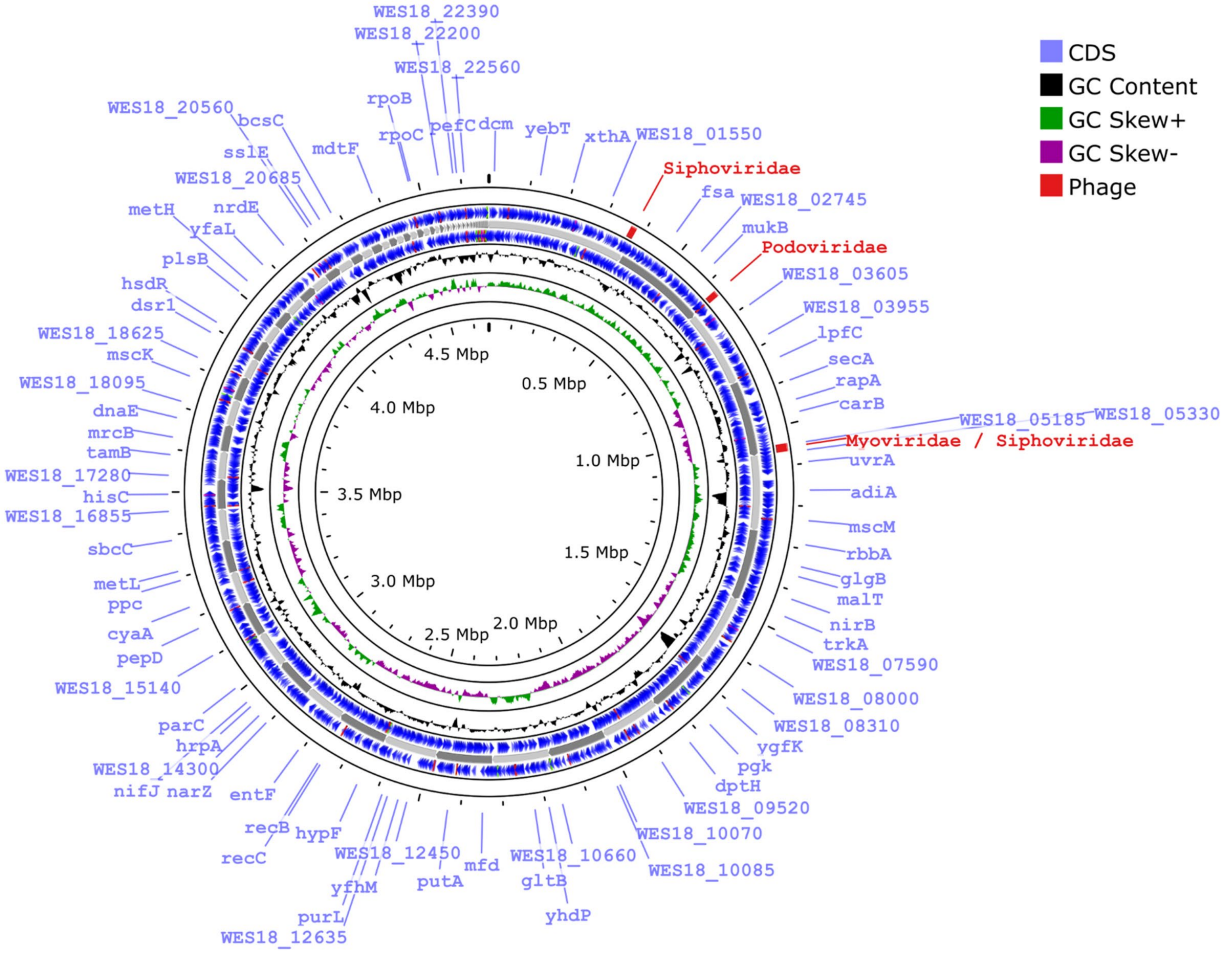
Genome map of Ea_FM1 with prophage elements denoted in red.

RAST annotation of the draft genome revealed the presence of 2,081 coding sequence regions with majority of them associated with carbohydrate metabolism (349/2,081, 16.77%) followed by amino acids metabolism (297/2,081, 14.27%) and protein metabolism (225/2,081, 10.81%) (Figure 3). Though, the isolate was found to be non-motile, 97 coding regions associated with motility and chemotaxis were identified in the genome. Snippy tool identified 20 instances of mutational events which could be impactful. Out of those 20 instances, 12 were of deletion type and 8 were of insertion type mutations (Supplementary Table 2).

### 3.5 Genomic repertoire of resistance determinants, virulence genes and mobile genetic elements

Identification of antimicrobial resistance determinants in the genome revealed the presence of 43 different resistance ontologies conferring resistance to various classes of antibiotics such as macrolides, aminoglycosides, cephalosporins, tetracyclines, peptides, nitroimidazoles, penams, fluoroquinolones, diaminopyrimidines, phenicols, glycopeptides, penems, glycylcyclines, monobactams, etc. (Table 2). Majority of the genes exert their action through antibiotic efflux mechanism (*n* = 32), followed by target alteration (*n* = 14), reduced permeability (*n* = 2) and inactivation (*n* = 1). On screening for virulence genes, a total of 113 virulence genes were identified (Table 3). Categorization the virulence genes into various virulence classes revealed that majority of the genes belonged to effector delivery system (*n* = 58), followed by nutritional/metabolic factor (*n* = 22), adherence (*n* = 17), regulation (*n* = 6), exotoxin (*n* = 4), invasion (*n* = 4), antimicrobial activity (*n* = 1) and immune modulation (*n* = 1). Pathogenic potential of the isolate toward humans was found to be on the higher side with a probability of 0.883. A total of three plasmids (IncFIA, IncFIB(pB171), IncFII(pSE11)) and two transposons (Tn*3411*, Tn*6292*) were identified from the genome; however, no integrons could be identified. Phigaro analysis detected prophages of three different phages inserted into the genome namely siphoviridae (12 virus orthologous groups), podoviridae (17 virus orthologous groups) and myoviridae/siphoviridae (23 virus orthologous groups) (Supplementary Table 3).

**Table 2:** Antimicrobial resistance genes observed in *Escherichia albertii* Ea_FM1 genome.

Antibiotic resistance ontology	Resistance mechanism
*Klebsiella pneumoniae KpnE*	Efflux
*Klebsiella pneumoniae KpnF*	Efflux
*Escherichia coli mdfA*	Efflux
*msbA*	Efflux
*leuO*	Efflux
*mdtP*	Efflux
*mdtN*	Efflux
CRP	Efflux
*Escherichia coli emrE*	Efflux
H-NS	Efflux
*AcrE*	Efflux
*mdtH*	Efflux
*mdtG*	Efflux
*acrD*	Efflux
*rsmA*	Efflux
*kdpE*	Efflux
*acrB*	Efflux
*Escherichia coli acrA*	Efflux
*mdtA*	Efflux
*mdtB*	Efflux
*mdtC*	Efflux
*emrB*	Efflux
*emrA*	Efflux
*emrR*	Efflux
*mdtF*	Efflux
*mdtE*	Efflux
*baeR*	Efflux
*marA*	Efflux; reduced permeability
*Escherichia coli UhpT* with mutation conferring resistance to fosfomycin	Target alteration
*Haemophilus influenzae* PBP3 conferring resistance to beta-lactam	Target alteration
*Escherichia coli* EF-Tu mutants conferring resistance to Pulvomycin	Target alteration
*Escherichia coli GlpT* with mutation conferring resistance to fosfomycin	Target alteration
*Escherichia coli soxS* with mutation	Target alteration; efflux; reduced permeability
*Escherichia coli soxR* with mutation	Target alteration; efflux
*Escherichia coli AcrAB-TolC* with *AcrR* mutation conferring resistance to ciprofloxacin, tetracycline, and ceftazidime	Target alteration; efflux
*Escherichia coli AcrAB-TolC* with *MarR* mutations conferring resistance to ciprofloxacin and tetracycline	Target alteration; efflux
*eptA*	Target alteration
*Escherichia coli* ampC beta-lactamase	Inactivation
*vanG*	Target alteration
*ugd*	Target alteration
*bacA*	Target alteration
*ArnT*	Target alteration
*PmrF*	Target alteration

**Table 3:** Virulence genes in the *Escherichia albertii* Ea_FM1 genome.

Virulence classes	Virulence genes
Effector delivery system (*n* = 58)	*nleA/espI, nleC, nleD, nleH1, nleH1, sepD, escC, escD, escF, escJ, escK, escP, escR, escS, escT, escU, escV, glrA, glrR, escI, sepQ/escQ, espH, sepL, espA, espB, espD, espF, cesAB, cesD, cesL, cesD2, escG, espJ, espK, map, hcp1/tssD1, tssA, gspC, gspD, gspE, gspF, gspG, gspH, gspI, gspJ, gspK, gspL, gspM, escN, escO, cesF, cesT, eae, escE, rorf1, etgA, escL, tssM*
Adherence (*n* = 17)	*fimA, fimB, fimE, fimI, fimF, fimG, fimH, fimC, fimD, paa, csgC, csgA, cgsE, cgsF, cgsG, cgsD, csgB*
Nutritional/metabolic factor (*n* = 22)	*chuA, fes, entF, entS, fepA, fepB, fepC, fepD, fepG, entD, fepE, entC, entE, entB, entA, shuV, chuU, chuY, chuX, chuW, chuT, chuS*
Exotoxin (*n* = 4)	*east1, cdtA, cdtB, cdtC*
Invasion (*n* = 4)	*ompA, ibeC, ibeB, aslA*
Regulation (*n* = 6)	*pmrA, rcsB, rpoS, fur, phoP, AAC38364*
Antimicrobial activity/competitive advantage (*n* = 1)	*acrB*
Immune modulation (*n* = 1)	*gndA*

### 3.6 Molecular epidemiology cues

**Figure 4:**
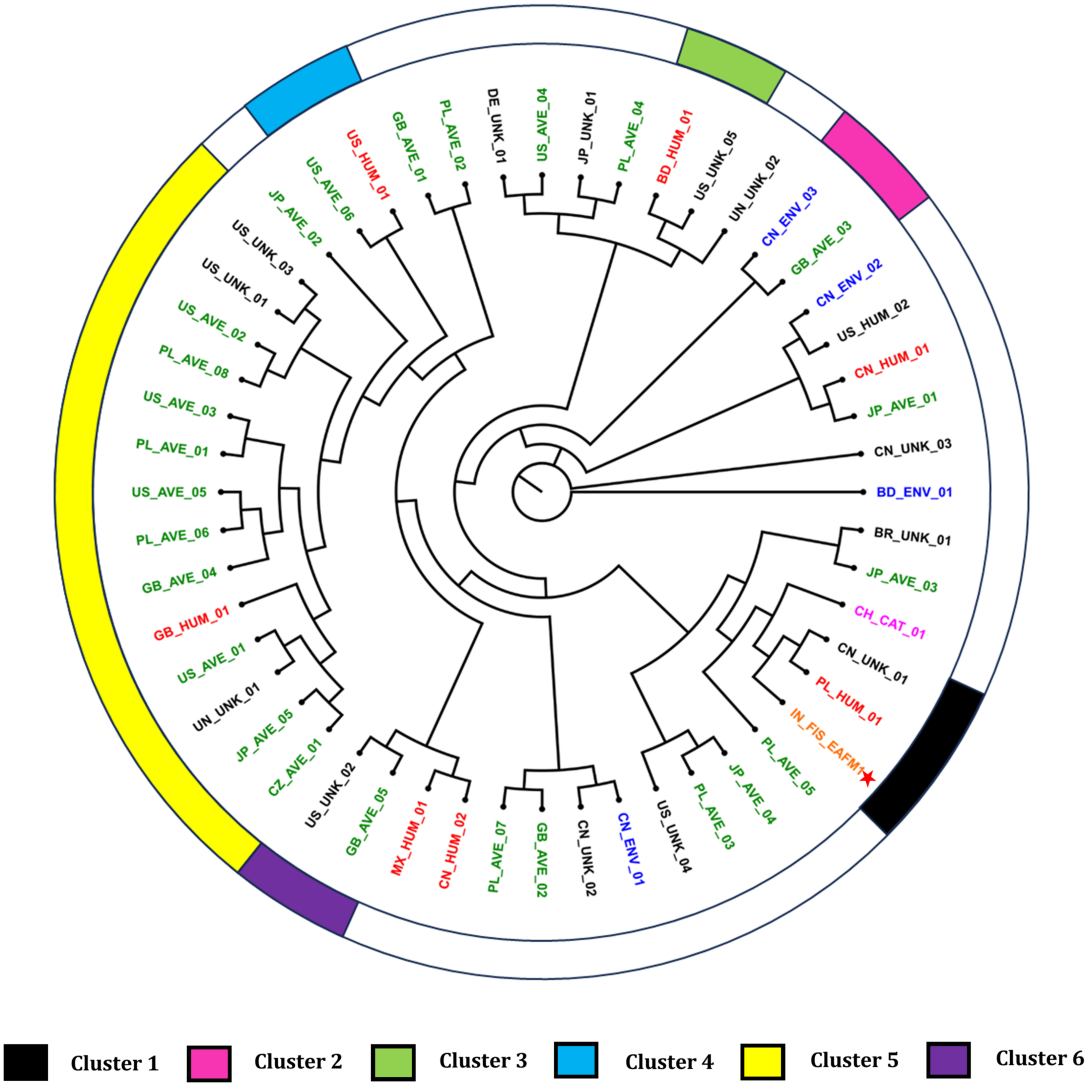
Phylogenomic tree of *E. albertii* genomes. Asterisk indicates the fish isolate Ea_FM1 sequenced in this study. The genomes were assigned codes X_Y_Z, where “X” indicates the country of isolation (ISO 3166-1 Alpha-2 code), “Y” indicates the source of isolation, and “Z” denotes the serial number of strain (i.e., BD, Bangladesh; BR, Brazil; CH: Switzerland; CN, China; CZ, Czech Republic; DE, Germany; GB, Great Britain; IN, India; JP, Japan; MX, Mexico; PL, Poland; US, United States of America; UN, unknown country of isolation; UNK, unknown source of isolation). Additionally, the coded genome names were given colors based on the source of isolation.

MLS typing using *E. coli* PubMLST scheme matched with sequence type 4,596 with alleles adk_387, fumC_111, gyrB_408, icd_483. mdh_109, purA_149 and recA_75. Further, O and H antigen-based typing revealed that the isolate belonged to H1 and O4 genotype. Core genome based phylogenomic analysis with GTR + F + I + R6 model placed the isolate Ea_FM1 in close proximity with a Chinese isolate of unknown isolation source and an isolate PL_HUM_01. (Figure 4). The clusters were identified with a cut-off value of 0.0010146631, as identified by the tool. A total of six clusters were identified with a Dunn index of 1.421.

## 4 Discussion

*E. albertii* is gradually reiterating its status as an emerging pathogen with an increase in spectrum in terms of geographical range, isolation source and clinical manifestations in humans (Muchaamba et al., 2022). Clinical infections implicated with *E. albertii* include watery to bloody diarrhea, fever, abdominal distension, dehydration and urinary tract infections (Muchaamba et al., 2022).

The reservoir of *E. albertii* are widely believed to be poultry, owing to its higher prevalence rates reported in various studies (Asoshima et al., 2015; Maeda et al., 2015; Wang et al., 2016; Liu et al., 2023a). However, its isolation from diverse sources such as pigs, dogs, bats, raccoons, penguins, and seals, combined with the absence of this organism in some poultry flocks, has made this claim debatable (Gordon, 2011; Wang et al., 2022; Muchaamba et al., 2022). As an enterobacterial pathogen, *E. albertii* is shed in the feces of animals, contaminating the environment and potentially re-entering the human food chain through contaminated food and water. Reports indicating its presence in leafy vegetables and foods of animal origin further support this hypothesis (Fiedler et al., 2018; Arai et al., 2022; Hirose et al., 2022; Muchaamba et al., 2022). Despite these findings, the transmission dynamics of *E. albertii* remain unclear, requiring further studies to identify its primary source. Additionally, its isolation from migratory birds provides valuable insights into the pathogen’s potential for spatial transmission across continents (Liu et al., 2023a; Barmettler et al., 2022; Carter et al., 2023).

The current study, being the first to report isolation of *E. albertii* from retail fish intended for consumption, has added a new entry into the list of isolation sources from which *E. albertii* has been isolated till date. Report from Japan on the prevalence in oysters was the closest isolation source reported from aquatic fauna (Arai et al., 2022). In addition to the earlier records of occurrence in various foods of animal origin, this addition highlights the importance of the pathogen from a food safety point of view and possible risk to fish eating population. It is to be noted that *E. albertii* was previously implicated in numerous food-borne outbreaks in Japan and has been infrequently isolated from water (Fiedoruk et al., 2014; Maheux et al., 2014; Masuda et al., 2020).

*Enterobacteriaceae* family has always been at the limelight in terms of antimicrobial resistance and has been placed in the WHO list of priority pathogens to inform research and development and public health interventions (World Health Organization, 2024). The ichthyic isolate showed phenotypic resistance to gentamicin (aminoglycoside), azithromycin (macrolide) and cephalosporins such as cefotaxime and ceftazidime. Incidentally, genomic characterization also revealed the presence of AMR genes known to confer resistance against these three classes of antibiotics. This phenotypic and genotypic correlation in terms of antimicrobial resistance was also evident in our previous study (Srinivas et al., 2023). Additionally, the isolate was also found to be multidrug-resistant which is of public health concern. MAR index of 0.33 (above the cut-off value of 0.2) hints at the probable exposure of the pathogen to environment rich in antibiotic pressure (Krumperman, 1983). Despite the presence of few earlier reports of biofilm forming *E. albertii*, the ichthyic isolate failed to produce biofilm on polystyrene surface (Ingle et al., 2011; Lima et al., 2019; Carter et al., 2024). Additionally, the ichthyic isolate was also found to be non-haemolytic and non-motile @ 37°C as reported earlier. Biotyping scheme as suggested by (Murakami et al., 2019) placed the isolate in Biotype 3 which is the predominant biotype in circulation as reported earlier (Murakami et al., 2019).

Mining for resistance genes identified the presence of AmpC beta-lactamase which is usually chromosomally encoded and rarely plasmid encoded (Jacoby, 2009). Presence of *ampC* gene among *E. albertii* isolates is not uncommon as it was identified to be the sole intrinsic beta-lactam resistance genes in all 12 isolates recovered from Wild birds in Switzerland (Barmettler et al., 2022). Genomic screening for virulence genes detected the presence of important virulence genes such as *cdt* and *astA*. The cytolethal distending toxin (CDT) coded by *cdt* gene is a AB2 toxin composed of subunits *cdtA*, *cdtB* and *cdtC* which results in distension of cells by disruption of the cell cycle (Scuron et al., 2016). CDT was also identified other clinically important pathogens such as *Campylobacter jejuni, Shigella dysenteriae, E. coli*, etc. (Scuron et al., 2016). The CDT present in *E. albertii* has been used as a diagnostic marker for the molecular confirmation due to the conserved nature of CDT type II gene (Hinenoya et al., 2019). Intimin coded by *eae* gene is an outer membrane protein which is responsible for the formation of attaching-effacing lesions on the epithelial cells (Romão et al., 2020). EAST1 encoded by *astA* gene shares 50% homology with heat stable STa toxin usually identified in Enterotoxigenic *E. coli* (ETEC) which can cause diarrhea in humans as well as animals (Dubreuil, 2019). Though not initially associated with *E. albertii*; recent reports have indicated presence of EAST1 among the virulence repertoires of *E. albertii* (Luo et al., 2021; Liu et al., 2023a). Pathogen Finder which calculates pathogenic potential, based on matching the protein families associated with disease causation, calculated a high probability for the isolate to be virulent toward humans (Cosentino et al., 2013). Analyzing the isolate using an animal model, such as *Caenorhabditis elegans*, would be intriguing. Notably, *E. albertii* is known to exhibit cytotoxic effects on various cell lines, including CHO, Vero, MDCK, and HeLa cells (Srinivas et al., 2024). Verotoxin producing strains of *E. albertii* could well lead to clinical manifestations such as Haemolytic Uraemic Syndrome, thrombotic microangiopathy and renal failure as identified in relation to verotoxin producing strains of *E. coli* (Banerjee et al., 2001; Muchaamba et al., 2022).

Mobile genetic elements in food borne pathogens have always been a matter of concern in terms of contaminating the food value chain and causing a health threat to the consumers (Partridge et al., 2018). Plasmids play an important role in harboring and dissemination of antimicrobial resistance genes, virulence genes and sometimes heavy metal resistance genes (Rodríguez-Beltrán et al., 2021). In this study, IncFIB(pB171) and IncFII(pSE11) are the most significant findings in terms of their ability to carry NDM genes as reported in earlier studies (Takayama et al., 2020; Hirabayashi et al., 2021). Transposons are genetic moieties which tend to jump from one genome to another and often carry resistance genes along with them (Babakhani and Oloomi, 2018). Tn*3411* identified in this study is a composite transposon which is known to effect citrate utilization (Ishiguro et al., 1982). The other transposon, Tn*6292* is a member of Tn*3*-family and often contained resistance genes against fluoroquinolones (Babakhani and Oloomi, 2018; Hua et al., 2021). Phage elements getting entangled in the genome of bacteria and the role played by them in the horizontal transfer of resistance genes is always a matter of concern (Rose et al., 2023). Siphoviridae, Myoviridae and Podoviridae exhibit a head and tail structure by virtue of their placement in order Caudovirales and they differ by means of the length and contractility of the tail region (Loh et al., 2020). These three phage taxons formed the predominant group of prophages in 177 *Acinetobacter baumannii* genomes with propensity to harbor AMR genes in a previous study (Loh et al., 2020).

Multi Locus sequence typing uses the polymorphism patterns of house-keeping genes to assign sequence type and closely bound sequence types form a clonal complex. In terms of *E. albertii,* MLST has been used for the identification of *E. albertii* and delineation from *E. coli* (Hyma et al., 2005). Adding to that, *E. albertii* does not have a scheme on its own and is always analyzed with schemes of *E. coli* (Luo et al., 2021; Barmettler et al., 2022). The sequence identified in this study was previously reported in a comparative genomics study from China in which the isolates with ST4596 were recovered from China and United Kingdom (Luo et al., 2021). Infrequent adoption of MLST analysis in various studies limits the utility of MLST as a suitable epidemiological tool in *E. albertii*. Somatic and flagellar antigen-based typing methods have been used in various bacteria to understand their epidemiology (van Belkum et al., 2007). Information on somatic “O” and flagellar “H” antigens provide sufficient discrimination about the serotypes of the outbreak and tracing the directions of the outbreak (Salazar et al., 2015; Sabat et al., 2013). Further, it also helps to understand the global epidemiology of the pathogen with a view to develop appropriate diagnostic tools and control measures (van Belkum et al., 2007). The EaOg4 type identified in this study was found to be the dominant type in a study from China (Liu et al., 2023b); however, it was absent in 47 *E. albertii* isolates recovered from migratory birds in China (Liu et al., 2023a). The flagellar antigen EaHg1 was identified in *E. albertii* isolate from the current study. However, the utility of these typing methods in the long run is yet to be ascertained.

Phylogenomic placement of the fish isolate close to the Chinese isolate could be justified because of the sharing of the land border and the rivers originating from China. Additionally, this could be attributed to the role of migratory birds as Indian subcontinent forms a major route for winter migration (Malik et al., 2021). The isolation and identification of *E. albertii* from migratory birds from China (Liu et al., 2023a), Switzerland (Barmettler et al., 2022) and USA (Carter et al., 2023) adds support to this argument that migratory birds can disseminate the organism. The fish isolate also clustered in close proximity with a human isolate from Poland. Additionally, human isolates clustering close to the avian isolates in various clusters highlights the zoonotic potential of *E. albertii*. However, elucidation of epidemiological links still needs efforts and further refinement. Based on our current understanding on the epidemiological mechanisms of the organism, adoption of strict biosecurity measures in the farms, early detection and hygiene food handling practices could serve as an important step to reduce the burden at farm level and subsequently reduce the risk of being transmitted along the food chain.

## 5 Conclusion

The current study is the first report of isolation and genomic characterization of *E. albertii* from fish origin. Presence of multidrug resistance and multiple toxin-producing virulence genes highlights the danger potential of *E. albertii* from a food safety point of view. Phylogenomic analysis revealed close clustering of the genome with a human isolate from Poland and a genome with unknown isolation source from China. Clustering of human isolates in close proximity with avian isolates provide cues on the mode and direction of transmission of this emerging pathogen which can serve as the starting point for future studies.

## Data Availability

The genome sequenced in the study was deposited in the NCBI repository, accession number JBBEFQ000000000.1 under BioProject: PRJNA889995.

## Footnotes

^1^ https://github.com/rrwick/Assembly-Dereplicator

^2^ https://github.com/tseemann/abricate

^3^ https://cge.food.dtu.dk/services/PathogenFinder/

^4^ https://cge.food.dtu.dk/services/PlasmidFinder/

^5^ https://github.com/bobeobibo/phigaro

^6^ https://vogdb.org/

^7^ https://cge.food.dtu.dk/services/MLST/

^8^ https://github.com/rambaut/figtree/releases
